# Development and Validation of an Instrument Measuring Theory-Based Determinants of Monitoring Obesogenic Behaviors of Pre-Schoolers among Hispanic Mothers

**DOI:** 10.3390/ijerph13060554

**Published:** 2016-06-02

**Authors:** Paul Branscum, Karina R. Lora

**Affiliations:** 1 Department of Health & Exercise Science, University of Oklahoma, Norman, OK 73019, USA; 2 Department of Nutritional Sciences, University of Oklahoma Health Sciences Center, Oklahoma City, OK 73019, USA; karina-lora@ouhsc.edu

**Keywords:** integrated behavioral model, childhood obesity, Hispanic health, health disparities

## Abstract

Public health interventions are greatly needed for obesity prevention, and planning for such strategies should include community participation. The study’s purpose was to develop and validate a theory-based instrument with low-income, Hispanic mothers of preschoolers, to assess theory-based determinants of maternal monitoring of child’s consumption of fruits and vegetables and sugar-sweetened beverages (SSB). Nine focus groups with mothers were conducted to determine nutrition-related behaviors that mothers found as most obesogenic for their children. Next, behaviors were operationally defined and rated for importance and changeability. Two behaviors were selected for investigation (fruits and vegetable and SSB). Twenty semi-structured interviews with mothers were conducted next to develop culturally appropriate items for the instrument. Afterwards, face and content validity were established using a panel of six experts. Finally, the instrument was tested with a sample of 238 mothers. Psychometric properties evaluated included construct validity (using the maximum likelihood extraction method of factor analysis), and internal consistency reliability (Cronbach’s alpha). Results suggested that all scales on the instrument were valid and reliable, except for the autonomy scales. Researchers and community planners working with Hispanic families can use this instrument to measure theory-based determinants of parenting behaviors related to preschoolers’ consumption of fruits and vegetables, and SSB.

## 1. Introduction

Pediatric obesity is one of the highest public health concerns in today’s society. In the United States, 8.4% of 2–5 year old children are obese [1]. Hispanic children are among those at greatest risk [2]. Hispanic children (2–5 years old) have the highest obesity prevalence of 16.7% in the nation, when compared to other racial and ethnic groups [1]. Health consequences associated with obesity range from metal health problems to metabolic abnormalities [3].

Public health obesity interventions that target young minority children are greatly needed to ameliorate the negative consequences associated with health disparities and medical costs for future generations. However to be effective, childhood obesity prevention efforts need to account for the inclusion of community members of the target group during all phases of program planning, involve schools and community resources to maximally leverage efforts, and be culturally appropriate [4]. At the microsystem level, parents are agents of change for children because they have the responsibility to shape their food environment and eating behaviors during each stage of development [5]. While parent-based childhood obesity prevention interventions report some success, more work is needed to assist parents in predisposing, enabling and reinforcing healthy behaviors to their children [6].

**Figure 1:**
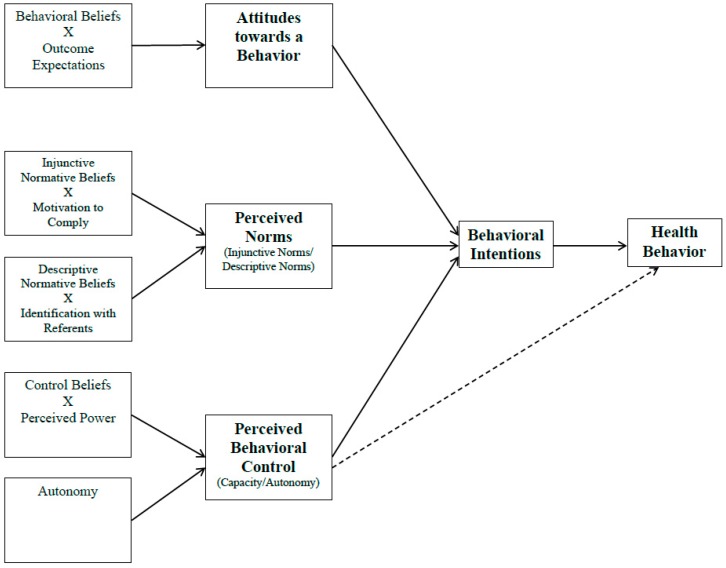
The Integrated Behavioral Model.

Determinants pertaining to how parenting behaviors influence children’s health behaviors have not been well established or reported in the literature. In that regard, health behavior theories that include social, environmental, and individual level factors can be operationalized and tested to better understand, explain and predict parenting behaviors related to key obesogenic behaviors in childhood, and provide a framework for public health interventions. While many health behavior theories and models exist, the Integrative Behavioral Model (IBM-Figure 1) was conceptualized and developed to integrate the leading theories and models being used in health behavior research [7]. Initially, the IBM contained eight principle determinates of health behaviors (intentions, environment, skills/abilities, attitudes, social pressure, personal standards/self-image, emotional reaction, and self-efficacy); however, since its inception little research has been done using the model. More recently, Martin Fishbein and Icek Ajzen utilized the principle determinants of the model within their reasoned action framework, to what is currently known as the IBM [8,9].

Instruments evaluating theory-based determinants of parenting behaviors related to obesogenic behaviors in childhood are greatly needed to facilitate more research in this critical area of investigation. Such instruments should be tested for validity and reliability, to assure that knowledge gained from research is trustworthy and holds a high level of scientific rigor. The purpose of this study was to develop an IBM-based instrument to evaluate maternal monitoring of fruit and vegetables, and sugar-sweetened beverages (SSB) with low-income Hispanic mothers of preschoolers, and to test its validity and reliability.

## 2. Methods

### 2.1. Planning Procedures for the IBM Instrument

Critical steps for program planning and instrument development were used to develop the theory-based instrument reported upon in this study [10]. First, nine focus groups were conducted in Spanish with Hispanic mothers (*n* = 55) of 2–5 year-old children. The mean age of the mother’s was 34.6 ± 8.0 years, and their reported place of birth was mostly Mexico (85%) followed by Central or South America (11%) and the U.S. (4%). Mothers reported living in the U.S. an average of 12.0 ± 6 years, and 89% indicated they spoke only Spanish or Spanish better than English. Focus groups were audio-recorded, transcribed in Spanish, translated into English, and coded and analyzed for behaviors mothers reported were most obesogenic for their children, and important for obesity prevention interventions. Overall, mothers reported 56 behaviors, within five broad categories: behaviors parents should do to help their children be healthy (*n* = 13 behaviors; example (ex.): Eat meals at an appropriate times); behaviors parents should do in the presence of other family members (*n* = 4 behaviors; example: Eat meals together as a family); purchasing behavior (*n* = 4 behaviors; example: Do not buy soda/SSB at the store); cooking behaviors (*n* = 8 behaviors; example: Prepare foods in a child-friendly manner (*i.e.*, cut fruit into flowers)); and parental monitoring behaviors (*n* = 27 behaviors; example: Have my children drink less soda/SSBs).

After examining the behaviors mothers reported from focus groups, it was apparent that while some behaviors were specific, most were vague. As Fishbein and Ajzen [9] note, research interested in predicting and changing health behaviors should begin with clearly defined behaviors, that contain an *action*, a *target* for the action, a *context* in which the action is performed, and a *time* period to perform the behavior. This is commonly known as the TACT principle of defining behaviors. Therefore, the 56 aforementioned behaviors were re-defined to include each element of TACT, resulting in a distillation to 38 TACT-specific behaviors, within the five broad categories of behaviors.

Next, each re-defined behavior was rated as “Low”, “Medium”, or “High” on its level of importance and changeability by the study investigators. Rating behaviors in this way is a common procedure in community organization and public health research (ex. the final step of the Epidemiological Assessment of the PRECEDE–PROCEED mode), to help community members and researchers focus on issues that can make the most impact on communities [11]. Through this process, two behaviors were identified as highly important and changeable. Behavior 1: Making sure my preschooler eats half of his/her plate filled with fruits and vegetables, at least 5 days a week (Spanish translation as it appeared on the instrument—Asegurarme de que mi hijo(a) coma la mitad de su plato de frutas y verduras por lo menos 5 días a la semana). Behavior 2: Make sure my preschooler does not drink sugary beverages (Kool-Aid^®^, Capri-Sun^®^, Soda, Tampico^®^) (Spanish translation as it appeared on the instrument - Asegurarme de que mi hijo(a) no beba bebidas azucaradas (Kool-Aid^®^, Capri-Sun^®^, Soda, Tampico^®^).

### 2.2. Development of the IBM Instrument

The IBM specifies that an individual’s intentions (or readiness) to perform a behavior is the most predictive determinants of a health behavior, and in turn, intentions are determined by the individual’s attitudes toward a behavior (or the overall evaluation of the advantages and disadvantages of a behavior), perceived norms (or social pressure to engage in a behavior), and perceived behavioral control (PBC; or how much control one feels over the behavior and how easy or difficult the behavior is to enact). Since the IBM is a *value-expectancy model*, the determinants of behavioral intentions can further be broken down for evaluation [9]. The term *value-expectancy model* refers to cognitive pathways in which the determinants of intentions are formed. For example, attitudes towards a behavior are shaped when individuals form a belief about the likelihood an outcome or attribute is associated with engaging in a specified behavior. This is also known as a behavioral belief, and items on instruments evaluating behavioral beliefs are typically evaluated on unipolar 7-point semantic differential scales ranging from 1 (not likely) to 7 (likely). After behavioral beliefs are formed, an individual will further place a value on each behavioral belief, also known as the outcome expectation. Outcome expectations are typically evaluated on unipolar 7-point semantic differential scales ranging from −3 (bad) to +3 (good). When a behavioral belief and outcome evaluation of an item are evaluated, the responses are multiplied to represent the attitude towards the behavior (−21 indicating a strong negative attitudes and +21 indicating a strong positive attitude) [9]. The remaining constructs of the IBM were evaluated in this same way.

Perceived norms is a dual construct that includes both injunctive norms and descriptive norms. Injunctive norms are determined by an individual’s injunctive normative beliefs, or a listing of the most important people in one’s life who would support or not support the individual to engage in a specified behavior, and are evaluated on unipolar 7-point semantic differential scales ranging from 1 (not likely) to 7 (likely). After injunctive normative beliefs are formed, an individual will further place a value on each injunctive normative belief, also known as the motivation to comply, which are typically evaluated on unipolar 7-point semantic differential scales ranging from −3 (should not) to +3 (should). Descriptive norms represent the newest development in the IBM, and has not been evaluated as frequently as injunctive norms. Like injunctive norms, descriptive norms are determined by descriptive normative beliefs, or a listing of people an individual considers would behave in a similar way, and are evaluated on unipolar 7-point semantic differential scales ranging from 1 (not likely) to 7 (likely). After descriptive normative beliefs are formed, an individual will further place a value each descriptive normative belief, also known as the identification with the referent, which are typically evaluated on unipolar 7-point semantic differential scales ranging from −3 (not at all) to +3 (very much).

The last determinant of intentions, PBC, is also a dual construct consisting of one’s autonomy and capacity towards action. Autonomy can only be measured directly, by asking participants how in control they feel they are towards performing a specified behavior. Capacity is determined by an individual’s control beliefs, or a listing factors that facilitate or impede one’s ability to perform a specified behavior, and are evaluated on unipolar 7-point semantic differential scales ranging from 1 (not likely) to 7 (likely). After control beliefs are formed, an individual will further place a value each control belief, also known as the perceived power, which is typically evaluated on unipolar 7-point semantic differential scales ranging from −3 (disagree) to +3 (agree).

To properly operationalize the IBM for the two behaviors identified through the focus groups, one-on-one interviews were conducted with Hispanic mothers (*n* = 20) in the community to elicit salient beliefs regarding the IBM constructs. The mean age of the mothers for the qualitative interviews was 34.3 ± 5.7 years. Additionally, 99% of the mothers reported being born in Mexico, 99% spoke only Spanish or Spanish better than English, and mothers had and average years living in the U.S. of 11.9 ± 5.1 years. A script was developed to elicit four specific types of beliefs through open-ended questions: Behavioral Beliefs (ex. What are some of the good things/bad things that could happen if you do the behavior?); Injunctive Normative Beliefs (ex. Who are important people in your life that want you/don’t want you to do this behavior?); Descriptive Normative Beliefs (ex. What people are most/least likely to do this behavior); and Control Beliefs (ex. What makes it easy/difficult for you to do this behavior?). Interviews were audio-recorded, transcribed in Spanish, and translated into English. Study investigators coded and analyzed interviews content for salient beliefs to develop items for the instrument.

For the behavior “Making sure my preschooler eats half of his/her plate filled with fruits and vegetables, at least 5 days a week” the highest ranking behavioral beliefs included “my child will” (1) be healthy; (2) develop properly; (3) get the appropriate nutrients; and (4) not become overweight. The highest ranking injunctive normative beliefs were (1) husband/partner; (2) grandparents of my preschooler; (3) and close family member; the highest ranking descriptive normative beliefs included (1) stay at home mothers; (2) close family members who also have preschoolers; and (3) other mothers who really care that their preschooler stays health. The highest ranking control beliefs included (1) when I encourage my preschooler; (2) when I pay close attention to what my preschooler eats; and (3) when I’m with my preschooler.

For the behavior “Make sure my preschooler does not drink sugary beverages (Kool-Aid^®^, Capri-Sun^®^, Soda, Tampico^®^)” the highest ranking behavioral beliefs included “my child will” (1) be healthy; (2) have less sugar; (3) not develop diabetes; and (4) not become overweight. The highest ranking injunctive and descriptive normative beliefs were the same as those identified for the fruit and vegetable behavior. The highest ranking control beliefs included (1) when others take care of my preschooler; (2) when I’m at home; and (3) when my preschooler nags me. All beliefs were used to develop the initial draft of the survey (for a copy of the survey in English or Spanish, please contact the corresponding author).

### 2.3. Measuring the Validity and Reliability of the IBM Instrument

Based on the one-on-one interviews, the instrument was developed and on the instrument, scales were formed to evaluate each construct of the IBM (intentions, attitudes, perceived norms, and PBC). Initially, the instrument was sent to a panel of six experts (two experts in instrument development; two experts in health behavior theory; two experts in Latino health) to establish face validity (whether the instrument appeared to measure what it was supposed to measure) and content validity (whether items had been adequately sampled within each scale to represent the entire meaning of the construct). After the panel submitted their initial suggestions, the instrument was revised and sent for a second round of review. Afterwards, the instrument was pilot tested with five Hispanic mothers of preschoolers, to elicit comments regarding the clarity of the items and ease of administration. Comments were used to further refine the instrument before data collection.

Psychometric testing of each scale on the instrument was conducted to establish its internal consistency reliability and construct validity. Internal consistency reliability was established using Cronbach’s alpha. For each scale, the following criteria was used to interpret the results: α > 0.8 was deemed *good*; 0.80 > α > 0.7 was deemed *acceptable*; 0.70 > α > 0.6 was deemed *questionable*; 0.60 > α > 0.5 was deemed *poor*; and an α < 0.5 was deemed *unacceptable* [12]. Scales deemed questionable, poor or unacceptable (α < 0.7), were re-specified by eliminating weak items that were detected using inter-item correlation matrices; unrelated items (*r* < 0.20) or redundant items (*r* > 0.80) to the scale were removed.

**Figure 2:**
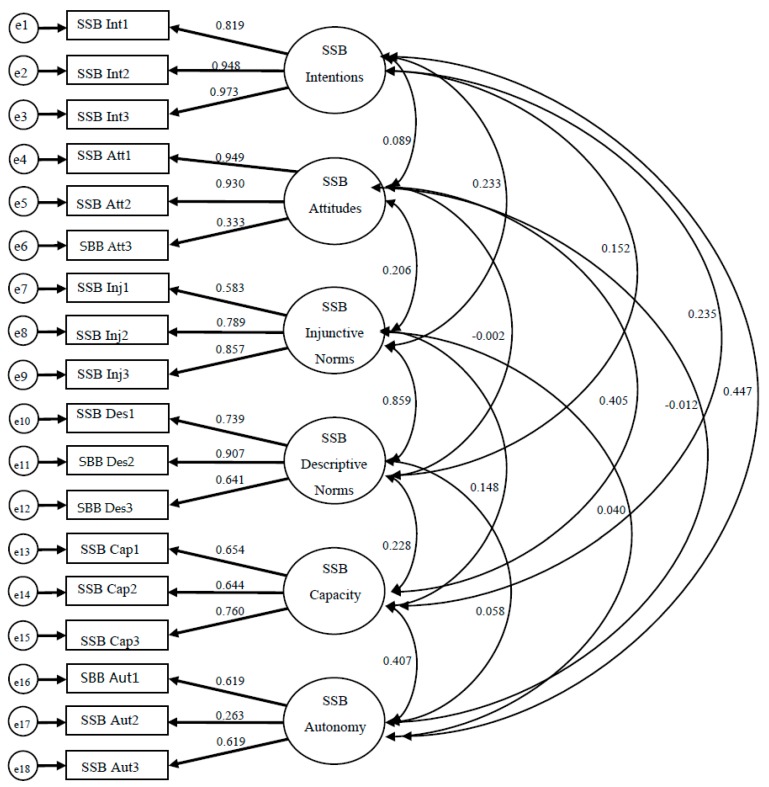
Results from confirmatory factor analysis (CFA) analyses for the sugar-sweetened beverage monitoring instrument (Comparative Fit Index (CFI) = 0.94, Tucker–Lewis Index (TLI) = 0.924, and Root Mean Square Error of Approximation (RMSEA) = 0.066).

**Figure 3:**
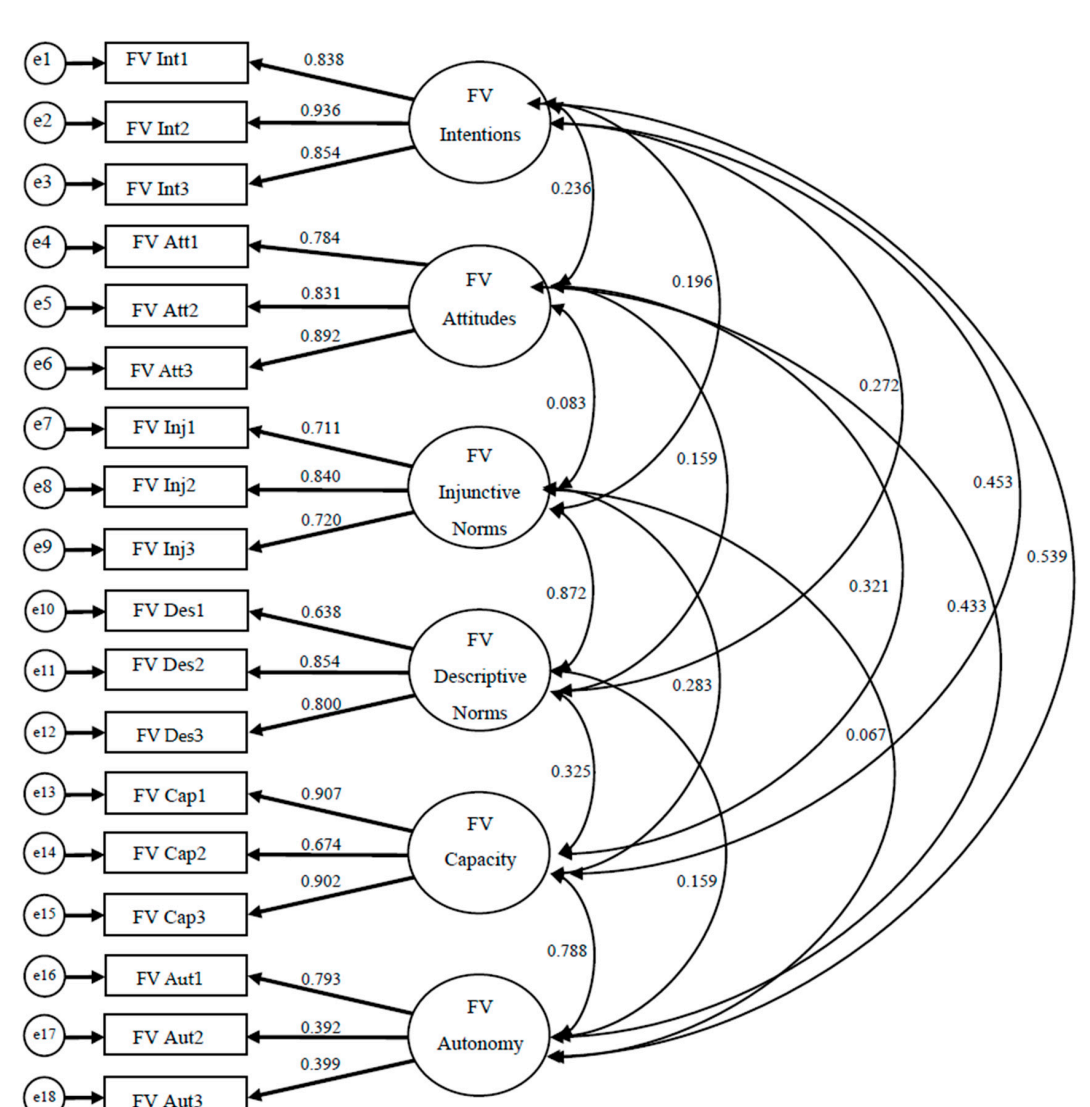
Results from CFA analyses for the fruit and vegetable monitoring instrument (FI = 0.972, TLI = 0.964, and RMSEA = 0.046).

To establish construct validity for each instrument, confirmatory factor analysis (CFA) was employed using the maximum likelihood method because data were normally distributed. Both instruments contained 19 items each, evaluating intentions (3 items), attitudes (4 items), injunctive norms (3 items), descriptive norms (3 items), autonomy (3 items), and capacity (3 items) for each behavior. Data were inspected for missing values, and in rare cases imputations were made for missing data. To employ CFA, we hypothesized a 6-factor model to be confirmed for each instrument’s measurement model (Figure 2 and Figure 3; Table 1 and Table 2). All exogenous variables for both models were allowed to covary. Instruments were deemed construct valid if items significantly loaded on the scale it was expected, and model fit indices were appropriate ((Comparative Fit Index (CFI ≥ 0.95), Tucker–Lewis Index (TLI ≥ 0.95), and Root Mean Square Error of Approximation (RMSEA ≤ 0.08)) [13]. All CFA analyses were done using SPSS AMOS (version 17) (IBM Analytics; Armonk, NY, USA).

In addition, a brief demographic questionnaire was developed and tested for understandability with five mothers of the target group. Participants’ acculturation was assessed using two questions that asked: number of years of residence in the U.S. and the language they generally read and speak (response options: only Spanish, Spanish better than English, Both equally, English better than Spanish, and only English) [14]. All of the instruments used for this study were initially created in English and translated into Spanish. The protocol for translating the interview guide and instruments from English to Spanish followed Marin’s double translation methodology [15]. To improve the likelihood that the translated documents would account for vocabulary variations in Spanish language of the target population (*i.e.*, country-specific words) the individuals that translated the study survey were from Mexico and Central America. Further, one of the study investigators (KL) who acted as a referee to compare the translated and back-translated versions, is a native Spanish speaker, bicultural, and has experience working with Hispanics from different countries. In addition, the translated version of the study survey was pilot tested for face validity with a small sample of participants of the target population (*n* = 5) to obtain information on question wording, understanding, and clarity.

Hispanic mothers were recruited from churches, community agencies, preschools, and daycares located in five zip codes in Oklahoma City, OK, where majority of the Hispanic population resides. Recruitment strategies included the use media (TV, radio, and newspaper advertisements), fliers, and a community health worker visit to recruitment sites to attract Hispanic families with 2-to-5-year-old children. A pre-screening was conducted to meet inclusion criteria of self-identification as Hispanic, living with a 2-to-5-year-old child, and the family being low income per Supplemental Nutrition Assistance Program eligibility criteria [16].

## 3. Results

Overall, the mean age of the Hispanic mothers (*n* = 238) was 33.1 years (±6.4) and their children was 3.8 years (±1.1). Almost half of the mothers were obese (42%) and slightly over a quarter of their children were obese (27%). Almost the entire sample (92%) was born outside of the U.S., and 80% spoke Spanish or Spanish more than English at home. Over half of the woman in this study were unemployed, and 73% attained a high-school education or less.

Upon examining the initial model fit indices and item-factor loadings for both instruments in the CFA analyses, it was determined that one item be removed from each of the attitudes scales. After this slight model re-specification, it was found that for the Fruit and Vegetable monitoring instrument, all scales yielded significant factor loadings, and good model fit indices (CFI = 0.972, TLI = 0.964, and RMSEA = 0.046) (Figure 3). Results for the Sugar-Sweetened Beverage monitoring instrument were similar, however two items on the Autonomy scale (Aut2 and Aut3) contained a factor loading that were not significant. However, for the model fit indices one was adequate (RMSEA = 0.066), while the others were slightly less than expected (CFI = 0.94, TLI = 0.924) (Figure 2). Standardized parameter estimates can be found on Figure 2 and Figure 3, and unstandardized estimates are presented on Table 1 and Table 2. A correlation matrix for latent variables is presented on Table 3.

In turn, the Cronbach’s alpha scores measuring each scale’s internal consistency reliability were acceptable, except for the autonomy scales, which was deemed poor for the fruit and vegetable behavior, and unacceptable for the sugary beverage behavior. A summary of the reliability statistics can be found on Table 4.

## 4. Discussion

The purpose of this study was to develop an IBM-based instrument, and evaluate its validity and reliability. This was accomplished by including community members, and through focus groups identifying which behaviors members of the target population thought were most important for obesity prevention. Focus groups findings revealed that although mothers could identify an array of behaviors, they mostly cited parental monitoring behaviors as most critical for obesity prevention. Monitoring is a parental controlling behavior that parents implement to keep track of children’s intake of less healthy foods and direct children towards healthy eating [17]. While there is an increasing body of literature reporting a significant association between childhood obesity and different types of parenting practices [18], research investigating determinants of specific parenting practices, such as what underlining behaviors facilitate or impede a parent to monitor their child’s intake or certain foods, select food for purchase or cook foods for the family are behaviors that are greatly needed to form the foundation of future public health interventions [18]. Concurrently, as Patrick and colleagues [19] note “there is a critical need to develop measures that address the range of parenting practices and how parenting practices may differ across obesogenic behavioral domain”.

Parenting behaviors, such as monitoring or feeding practices, should not be confused with the concept of *parenting styles*, which is based upon Baumrind’s [20] and Maccoby and Martin’s [21] typology categorizing parents into one of four parenting styles (authoritarian, authoritative, permissive and uninvolved/neglectful). Parenting style refer to a global disposition parents have, and are used to make decisions as to how they raise their children. Within the context of the IBM, parenting style is viewed as a background factor that has the potential to directly influence a parent’s beliefs (ex. behavioral beliefs or control beliefs) pertaining to parenting practices and behaviors (*i.e.*, parental monitoring), but not directly influence the parent’s behaviors.

The second purpose of this article was to evaluate the validity and reliability of an IBM-based instrument, operationalized for parental monitoring of fruits and vegetables and sugary drinks. Overall, the instruments were found to have sufficient validity and reliability, except for the autonomy scales for both behaviors. This was surprising, since the instrument had a strong theoretical framework, was evaluated for content and face validity, and pilot tested with small sample of the target population before administration. It is possible that the items measuring autonomy had little variability, and this could deflate the inter-item correlations between items, and in turn, reduce the reported Cronbach’s alpha. A revision for the autonomy scale in both instruments is warranted.

A strength of this instrument is the strong theoretical basis for which it was developed, which can provide a guiding framework of public health interventions by specifying significant social, environmental, and individual level factors related to health behavior change. A recent review of the literature by a 24-member expert advisory group found that in the field of behavior change, there are 83 theories, of which the most popular were Transtheoretical Model, the Theory of Planned Behavior, Social Cognitive Theory, and the Information-Motivation-Behavioral Skills Model [22]. The IBM contains elements from all of these theories, and presents them in a parsimonious fashion, which is greatly needed for planning public health interventions. Another strength was the unique way descriptive norms was evaluated. While there is a long history of researchers who have operationalized the major constructs of the IBM (ex. attitudes, injunctive norms and PBC, according to Fishbein and Ajzen [9] (p. 148)), to date no study has operationalized descriptive norms, using the value-expectancy model presented in this study.

Besides the strong theoretical basis for which this survey was developed, another strength to this study is how community planning was utilized. Community planning and organization has been defined in many ways, but the key principle lies in assisting communities for helping themselves, rather than relying on public health professionals to help them [11]. This is referred to as a bottom-up approach, rather than a top-down approach. To this end, the two behaviors used in this study were behaviors mothers in the community deemed important. While not a prerequisite, the behaviors the mothers in this community chose does have research supporting its link to obesity. While there are numerous determinants of childhood obesity, overconsumption of SSB and an inadequate amount of fruit and vegetable consumption are oftentimes noted as especially obesogenic. For example, in the United States an expert committee comprised of the American Medical Association and other leading health organizations, identified four behaviors health professionals should monitor and intervene upon for children, and the only two behaviors related to diet focused on fruit and vegetable consumption and sugar-sweetened beverage consumption [23]. Furthermore, research supports these two behaviors for Hispanic families [24], and suggests more work should be done intervening on how parents monitor health behaviors among children [25].

The final strength of this study lies in the process by which this instrument was developed. In this area of public health valid and reliable instruments are greatly needed, especially those that are tailored to specific communities. While many surveys and instruments have been developed, none could have evaluated the specific behaviors and beliefs pertaining to this community. To this end, while a number of community planning models call for evaluating determinants of health behaviors (*i.e.*, Step 3 of the PRECEDE–PROCEED model (the Educational and ecological assessment)) to develop targeted and tailored approaches for public health interventions, this could not have been accomplished without the development and validation of instrument [11].

This study has a few notable limitations that should be addressed. First, all of the responses on both instruments were based on self-reporting. Participant’s responses may have been biased and the beliefs reported might not truly represent their actual beliefs. Further, participants may have misinterpreted questions and provided erroneous answers. Second, the sample used for this study was from a convenience sample of Hispanic mothers with preschoolers, therefore the results from this study may not be generalizable to other racial and ethnic groups. Also, since this was a convenience sample, we were not able to control for national origin or acculturation of the participants, which can be related to their personal identity and influence their child-monitoring behavior. Future studies should consider evaluating these two variables, which can be treated as a background factor in the IM. Third, while many forms of validity and reliability are reported, test-retest reliability (or stability) was not established. Future researchers should consider establishing this.

## 5. Conclusions

In conclusion, the two behaviors studied in this article were parental monitoring for fruits and vegetables and sugary beverages, which represents the interpersonal level of the ecological model, which is commonplace in public health research and practice. Health practitioners and researchers should further utilize the ecological model when planning obesity prevention interventions, and include the other levels. For example, schools can encourage children to drink less sugary beverages and consume more fruits and vegetables as part of health education (an intrapersonal factor), parents can closely monitor the amount of SSBs and fruits and vegetables they allow their children to consume (an interpersonal factor), and schools (organizational factor) and communities (community factor) can implement policies to support behavior change by making clean water, and other sugar-free drinks, and fruits and vegetables, available and accessible. Concurrently, research evaluating theory-based determinants of behavior change using valid and reliable instruments is greatly needed. For example, in a recent review of seven journals in the field of health promotion and education (ex. American Journal of Health Behavior; Health Education and Behavior) authors examined nearly 1000 published studies and found that a high percentage failed to report measures of validity and reliability [26].

## Figures and Tables

**Table 1:** Standardized (**β**) and unstandardized (**B**) coefficients for CFA analysis for sugar-sweetened beverage (SSB) monitoring instrument.

Observed Variable	Latent Variable	β	B	SE
I intend to do this behavior every time I feed my preschooler (SSB Int1 *)	SSB Intentions	0.82	1	
I will try to do this behavior every time I feed my preschooler (SSB Int2 *)	SSB Intentions	0.95	1.19	0.06
I will to do this behavior every time I feed my preschooler (SSB Int3 *)	SSB Intentions	0.97	1.22	0.06
Doing this behavior will help my preschooler…				
Not become overweight * My preschooler being overweight is <Extremely Bad/Extremely Good> ^a^ (SSB Att1 *)	SSB Attitudes	0.95	1	
Not develop diabetes * My preschooler developing diabetes is <Extremely Bad/Extremely Good> (SSB Att2 *)	SSB Attitudes	0.93	1.03	0.07
Have less sugar * My preschooler having less sugar is <Extremely Bad/Extremely Good>(SSB Att3 *)	SSB Attitudes	0.33	0.33	0.07
My husband/partner thinks I should do this * When it comes to feeding my preschooler	SSB Injunctive Norms	0.58	1	
I want to do what my husband/partner thinks I should do. (SSB Inj1 *)				
The grandparents of my preschooler thinks I should do this * When it comes to feeding my preschooler I want to do what the grandparents of my preschooler thinks I should do. (SSB Inj2 *)	SSB Injunctive Norms	0.79	1.36	0.15
My close family members thinks I should do this*When it comes to feeding my preschooler. I want to do what my close family members thinks I should do. (SSB Inj3 *)	SSB Injunctive Norms	0.86	1.40	0.16
Most stay at home mothers who do not work would do this * When it comes to feeding my preschooler I want to be like other stay at home mothers who also have preschoolers. (SSB Des1 *)	SSB Descriptive Norms	0.74	1	
My close family members who also have preschoolers would do this * When it comes to feeding my preschooler I want to be like my close family members who also have preschoolers (SSB Des2 *)	SSB Descriptive Norms	0.91	1.12	0.09
Other mothers who really care that their preschooler stays healthy would do this * When it comes to feeding my preschooler I want to be like other mothers who really care that their preschooler is healthy. (SSB Des3 *)	SSB Descriptive Norms	0.64	0.94	0.10
My preschooler gets sugary drinks if other people take care of him/her * If my preschooler is being taken care of by other people it makes harder. (SSB Cap1 *)	SSB Capacity	0.65	1	
If I’m at home, I give my preschooler sugary drinks * Being home with my preschooler most of the time makes it easier to do this behavior. (SSB Cap2 *)	SSB Capacity	0.64	0.90	0.13
My preschooler nags me about giving him/her sugary drinks * If my preschooler nags me about giving him/her sugary drinks it makes it harder for me to do this behavior. (SSB Cap3 *)	SSB Capacity	0.76	1.19	0.14
It is up to me for making sure my preschooler does this. (SSB Aut1 *)	SSB Autonomy	0.62	1	
How much control do you have for making sure your preschooler does this <No Control/Total Control> (SSB Aut2)	SSB Autonomy	0.26	0.28	0.23
Making sure my preschooler does this behavior is <Not up to my/Totally up to me> (SSB Aut3)	SSB Autonomy	0.36	0.49	0.37

Note: Behavior for Instrument Make sure my preschooler does not drink sugary beverages (Kool-Aid^®^, Capri-Sun^®^, Soda, Tampico^®^); * *p*-value < 0.001; ^a^ Indicates reverse coding of item on scale.

**Table 2:** Standardized (**β**) and unstandardized (**B**) coefficients for CFA analysis for fruit and vegetable (F/V) monitoring instrument.

Observed Variable	Latent Variable	β	B	SE
I intend to do this behavior every time I feed my preschooler (FV Int1 *)	F/V Intentions	0.84	1	
I will try to do this behavior every time I feed my preschooler (FV Int2 *)	F/V Intentions	0.94	1.12	0.062
I will to do this behavior every time I feed my preschooler (FV Int3 *)	F/V Intentions	0.85	1.03	0.064
Doing this behavior will help my preschooler…				
Be healthy * My preschooler being healthy is <Extremely Bad/Extremely Good> (FV Att1 *)	F/V Attitudes	0.78	1	
Develop properly * My preschooler developing properly is <Extremely Bad/Extremely Good> (FV Att2 *)	F/V Attitudes	0.83	1.19	0.089
Get all the nutrients he/she needs * My preschooler getting the nutrients he/she needs is <Extremely Bad/Extremely Good> (FV Att3 *)	F/V Attitudes	0.89	1.15	0.084
My husband/partner thinks I should do this * When it comes to feeding my preschooler	F/V Injunctive Norms	0.71	1	
I want to do what my husband/partner thinks I should do. (FV Inj1 *)				
The grandparents of my preschooler thinks I should do this * When it comes to feeding my preschooler I want to do what the grandparents of my preschooler thinks I should do. (FV Inj2 *)	F/V Injunctive Norms	0.84	1.10	0.10
My close family members thinks I should do this*When it comes to feeding my preschooler	F/V Injunctive Norms	0.72	1.03	0.10
I want to do what my close family members thinks I should do. (FV Inj3 *)				
Most stay at home mothers who do not work would do this * When it comes to feeding my preschooler I want to be like other stay at home mothers who also have preschoolers. (FV Des1 *)	F/V Descriptive Norms	0.64	1	
My close family members who also have preschoolers would do this * When it comes to feeding my preschooler I want to be like my close family members who also have preschoolers (FV Des2 *)	F/V Descriptive Norms	0.85	1.32	0.13
Other mothers who really care that their preschooler stays healthy would do this * When it comes to feeding my preschooler I want to be like other mothers who really care that their preschooler is healthy. (FV Des3 *)	F/V Descriptive Norms	0.80	1.22	0.13
I my preschooler to do this behavior * Encouraging my preschooler makes it easier for me to do this. (FV Cap1 *)	F/V Capacity	0.67	1	
I pay close attention to what my preschooler eat when I am with him/her * Paying close attention to what my preschooler eat makes it easier for me to do this. (FV Cap2 *)	F/V Capacity	0.91	1.04	0.09
I plan to be with my preschooler when he/she eats meals when possible * Being with my preschooler	F/V Capacity	0.90	1.09	0.09
When he/she eat meals makes it easier for me to do this. (FV Cap3 *)				
It is up to me for making sure my preschooler does this. (FV Aut1 *)	F/V Autonomy	0.79	1	
How much control do you have for making sure your preschooler does this <No Control/Total Control> FV Aut2 *)	F/V Autonomy	0.39	0.50	0.10
Making sure my preschooler does this behavior is <Not up to my/Totally up to me> (FV Aut3 *)	F/V Autonomy	0.40	0.45	0.08

Behavior for instrument: Making sure my preschooler eats half of his/her plate filled with fruits and vegetables, at least 5 days a week; * *p*-value < 0.001.

**Table 3:** Pairwise correlation analysis of the latent variables.

SSB Instrument							
FV Instrument		**1**	**2**	**3**	**4**	**5**	**6**
	Intentions	-	0.089	0.233	0.152	0.235	0.447
	Attitudes	0.236	-	0.206	−0.002	0.405	−0.012
	In. Norms	0.196	0.083	-	0.859	0.148	0.040
	Des. Norms	0.272	0.159	0.872	-	0.228	0.058
	Capacity	0.453	0.321	0.283	0.325	-	0.407
	Autonomy	0.539	0.433	0.067	0.159	0.788	-

In. Norms (Injunctive Norms); Des. Norms (Descriptive Norms).

**Table 4:** A summary of the reliability statistics.

Theoretical Construct	Cronbach’s α
Fruit and Vegetables (F/V)	
Intentions	0.91
Attitudes towards the behavior	0.87
Injunctive Norms	0.80
Descriptive Norms	0.80
Capacity	0.85
Autonomy	0.59
Sugar Sweetened Beverages (SSB)	
Intentions	0.94
Attitudes towards the behavior	0.76
Injunctive Norms	0.79
Descriptive Norms	0.80
Capacity	0.72
Autonomy	0.41
